# L-Selectin Enhanced T Cells Improve the Efficacy of Cancer Immunotherapy

**DOI:** 10.3389/fimmu.2019.01321

**Published:** 2019-06-12

**Authors:** H. Angharad Watson, Ruban R. P. Durairaj, Julia Ohme, Markella Alatsatianos, Hanan Almutairi, Rebar N. Mohammed, Miriam Vigar, Sophie G. Reed, Stephen J. Paisey, Christopher Marshall, Awen Gallimore, Ann Ager

**Affiliations:** ^1^Division of Infection and Immunity, School of Medicine, Cardiff University, Cardiff, United Kingdom; ^2^PET Imaging Centre, School of Medicine, Cardiff University, Cardiff, United Kingdom; ^3^Systems Immunity Research Institute, Cardiff University, Cardiff, United Kingdom

**Keywords:** L-selectin/CD62L, T cells, melanoma, adoptive T cell therapy, cancer immunotherapy

## Abstract

The homing molecule, L-selectin (CD62L), is commonly used as a T cell activation marker, since expression is downregulated following engagement of the T cell receptor. Studies in mice have shown that CD62L^+^ central memory T cells are better at controlling tumor growth than CD62L^−^ effector memory T cells, while L-selectin knockout T cells are poor at controlling tumor growth. Here, we test the hypothesis that T cells expressing genetically modified forms of L-selectin that are maintained following T cell activation (L-selectin enhanced T cells) are better at controlling tumor growth than wild type T cells. Using mouse models of adoptive cell therapy, we show that L-selectin enhancement improves the efficacy of CD8^+^ T cells in controlling solid and disseminated tumor growth. L-selectin knockout T cells had no effect. Checkpoint blockade inhibitors synergized with wild type and L-selectin enhanced T cells but had no effect in the absence of T cell transfers. Reduced tumor growth by L-selectin enhanced T cells correlated with increased frequency of CD8^+^ tumor infiltrating T cells 21 days after commencing therapy. Longitudinal tracking of Zirconium-89 (^89^Zr) labeled T cells using PET-CT showed that transferred T cells localize to tumors within 1 h and accumulate over the following 7 days. L-selectin did not promote T cell homing to tumors within 18 h of transfer, however the early activation marker CD69 was upregulated on L-selectin positive but not L-selectin knockout T cells. L-selectin positive and L-selectin knockout T cells homed equally well to tumor-draining lymph nodes and spleens. CD69 expression was upregulated on both L-selectin positive and L-selectin knockout T cells but was significantly higher on L-selectin expressing T cells, particularly in the spleen. Clonal expansion of isolated L-selectin enhanced T cells was slower, and L-selectin was linked to expression of proliferation marker Ki67. Together these findings demonstrate that maintaining L-selectin expression on tumor-specific T cells offers an advantage in mouse models of cancer immunotherapy. The beneficial role of L-selectin is unrelated to its' well-known role in T cell homing and, instead, linked to activation of therapeutic T cells inside tumors. These findings suggest that L-selectin may benefit clinical applications in T cell selection for cancer therapy and for modifying CAR-T cells to broaden their clinical scope.

## Introduction

Immunotherapy represents a paradigm shift in cancer treatment; instead of directly attempting to kill cancerous tissue through radio- or chemotherapy, the patient's immune system is targeted, with the aim of augmenting the anti-tumor immune response. This may be done via systemic delivery of monoclonal antibodies that inhibit immune checkpoints exploited by tumors to induce immune tolerance, such as α-PD-1 (pembrolizumab, nivolumab) (1) or α-CTLA-4 (ipilimumab) (2). These pioneering immunotherapies are effective alone but behave synergistically to produce the most pronounced clinical effects when used together (3). The clinical outcomes of this approach have been dramatic, but the range of cancers amenable to checkpoint inhibition is limited and is thought to rely heavily on the cancer having a high neoantigen load (4). Many cancers, however, have only low to medium somatic mutation rates, leading to a limited range of neoantigens (4). Furthermore, T cells that are specific for cancer antigens often have only low affinity T cell receptors (TCRs) (5), limiting the efficacy of the endogenous T cell response, even in the presence of checkpoint inhibition.

To circumvent this, another class of immunotherapy involves adoptive transfer of T cells (adoptive cell therapy, ACT) into the patient. Initially, this approach was based on recovering tumor-infiltrating T cells (TILs) from resected cancers, expansion *ex-vivo*, and then re-infusion back to the patient. However, as understanding of the interaction between immune cells and tumor cells has increased, this approach has expanded to include a number of different strategies. Chimeric antigen receptor T (CAR-T) cells circumvent the need for a high-affinity tumor antigen-specific TCR by replacing the extracellular portion of the TCR with a monoclonal antibody receptor. This entirely bypasses the standard antigen presentation pathway, allowing CAR-T cells to be selectively targeted to the tumor tissue. However, to date, most CAR-T cells have been used to treat circulating hematological malignancies (6). Applying this technology to solid tumors has proved challenging, with one key obstacle being trafficking of T cells into tumors that are poorly vascularized (7, 8). The complexity of the tumor microenvironment means that some therapeutic approaches that directly target the tumor can also indirectly improve immune responses. Anti-angiogenic therapies were designed to induce tumor death via hypoxia. However, inhibiting rapid and abnormal angiogenesis had the unexpected effect of normalizing the tumor vasculature (9), and thus allowing improved infiltration of both chemotherapeutic agents and immune cells into the tumor. Indeed, the complexity of cross-talk between the immune system and cancer vasculature is only now being fully appreciated (10).

The leucocyte adhesion molecule L-selectin (CD62L) is a type-I transmembrane lectin receptor expressed on the surface of leucocytes. It is part of the selectin family, which also includes E-selectin (CD62E), expressed on endothelial cells, and P-selectin (CD62P), which is expressed on platelets and activated endothelium. L-selectin regulates entry of naïve and central memory T cells into lymph nodes and activated CD8^+^ T cells to sites of virus infection (11); L-selectin expression is regulated via proteolytic shedding of the ectodomain and transcriptional silencing of the L-selectin gene (12, 13). Downregulation of L-selectin on T cells is known to take place following engagement of the TCR, and this has led to L-selectin being used as a marker of T cell activation. It has been demonstrated that T cells with a naïve/central memory CD62L^+^ phenotype are better at controlling solid tumor growth than CD62L^−^ cytotoxic T cells (CTLs) in mice (14, 15). However, even when matched for differentiation status, T cells from L-selectin knockout mice are less effective than wild type T cells suggesting a direct role for L-selectin in controlling tumor growth (14). We hypothesize that cells which are genetically engineered to express high levels of mutant L-selectin which cannot be shed or gene-silenced would offer improved control of solid tumor growth, through increased T cell recruitment and infiltration of the tumor stroma. In this mutant L-selectin, the membrane-proximal region of CD62L has been replaced with the proteolysis-resistant membrane-proximal region of CD62P. We have therefore designated this modification as LΔP-selectin. In addition, the modified LΔP-selectin is expressed under a human CD2 promoter and locus control region which abrogates transcriptional silencing (16, 17). This study addresses whether these novel constructs serve to improve T cell-based cancer immunotherapy for solid and disseminated tumors.

## Materials and Methods

### Mice

Genotypes of mouse strains used in the study are shown in Table 1. C57BL/6J (B6) mice were purchased from Charles River, congenic B6.PL-Thy1a/CyJ mice, designated Thy1.1 hereafter, and FoxP3^DTR^ mice, supplied by Alexander Rudensky and maintained as previously described (18), were bred in house. L-selectin transgenic mice expressing wild type murine L-selectin (CD62Lwt) or a non-cleavable mutant L-selectin (CD62LΔP) on a B6 L-selectin^−/−^ background have been described (16, 19). F5 TCR transgenic mice generated as described previously (20), were backcrossed to B6 mice and maintained as a homozygous colony (F5B6). F5B6 mice were backcrossed to B6 L-selectin^−/−^ mice and maintained as a homozygous colony (F5LselKO). CD62LΔP mice were backcrossed to F5LselKO and maintained as a homozygous colony (F5LΔP). Hemizygous F5 mice were generated by crossing F5B6 with B6 mice and the F_1_ generation (F5/B6) used. T-cell donors and tumor-bearing hosts were matched for gender. Mice were housed in a specific pathogen free facility and allowed free access to water and food. Experimental mice were housed in individually ventilated cages. All mouse experiments conformed to the British Home Office Regulations [Animal (Scientific Procedures) Act, 1986 (Project Licenses PPL30/3188 and 30/2635 to AA)] and the protocol was approved by the Animal Welfare and Ethical Review Body at Cardiff University.

**Table 1:** Mouse genotypes.

**Mouse ID**	**Background**	**F5 TCR**	**Endogenous L-selectin**	**CD62L transgene**	**Thy**	**Tumor bearing host or T cell donor**
C57BL/6 (B6)	B6	No	+/+	No	Thy1.2	Host
Thy1.1	B6	No	+/+	No	Thy1.1	Host
FoxP3^DTR^	B6	No	+/+	No	Thy1.2	Host
CD62Lwt	B6	No	−/−	Wildtype hemizygous	Thy1.2	Host
CD62LΔP	B6	No	−/−	LΔP hemizygous	Thy1.2	Host
F5B6	B6	Homozygous	+/+	No	Thy1.2	Donor
F5LselKO	B6	Homozygous	−/−	No	Thy1.2	Donor
F5LΔP	B6	Homozygous	−/−	LΔP homozygous	Thy1.2	Donor
F5/B6	B6	Hemizygous	+/+	No	Thy1.2	Donor

### Adoptive T Cell Therapy

CD8^+^ T cells were isolated from pooled splenocytes and lymph nodes of naïve mice using a CD8a^+^ T cell isolation kit for negative selection, and LS columns, according to the manufacturer's instructions (StemCell Technologies) from 10.00 to 12.00 GMT on the day of injection. Briefly, spleens and/or lymph nodes were harvested from adult mice and mashed through a 70 μm cell strainer (BD Pharmingen). Red blood cells were lysed using red cell lysis buffer (Biolegend) and lymphocytes washed with ice-cold phosphate buffered saline (PBS) supplemented with 2% fetal calf serum (FCS) prior to magnetic isolation. The enriched CD8a^+^ cell fraction was counted using a hemocytometer, resuspended in sterile PBS for injection and analyzed for CD8, CD62L, CD44, CD27, and F5 TCR expression.

CTLs were generated as described previously (11). Briefly, CD8^+^ T cells from naïve mice (2 × 10^6^ cells/well) in 24-well plates (Nunclon) in complete medium (DMEM supplemented with 10% FCS, penicillin-streptomycin, L-glutamine, non-essential amino-acids and 2-mercaptoethanol) were incubated with 6 × 10^6^ NP68-peptide (ASNENMDAM; Peptide Synthetics) pulsed irradiated splenocytes (5 μM NP68 peptide for 1 h at 37°C, irradiated at 3,000 cGy), and plates incubated at 37°C in 5% CO_2_. Fresh complete medium supplemented with 360 IU/ml hrIL-2 (Proleukin, Chiron Ltd, UK) was added after 2 days and replaced with fresh medium on day 5. CTLs were harvested at day 7. Media was also supplemented with 25 ng/ml mu IL-15 (Peprotech, UK) for T cell cultures beyond 7 days.

### Tumor Cell Lines

The NP68-B16 melanoma cell line was generated as described previously (21, 22) and used to study adoptive T cell immunity to tumors in mice. Cells were validated for NP68 expression and confirmed mycoplasma free. In some experiments, the parental B16F10 melanoma cell line (ATCC number CRL-6475) was used to study endogenous T cell dependent immunity to tumors, as described previously (23).

### Solid Tumor Model

Frozen B16F10 or NP68-B16 cells were thawed from liquid nitrogen and cultured until second passage reached 100% confluence. Upon confluence, the cells were dislodged using StemPro Accutase (ThermoFisher Scientific) at 37°C and resuspended at 5 × 10^5^ cells in 200 μl sterile PBS. NP68-B16 cells were injected subcutaneously into the shaven left flank of female B6, Thy1.1, or Foxp3DTR mice. B16F10 cells were injected subcutaneously into the shaven left flank of female B6, CD62Lwt, or CD62LΔP mice. Mice were randomized into treatment groups for each experiment of 8–10 mice per group, tumor cells injected at 13.00–15.00 GMT and mice returned to their home cage. Tumors were measured every 3–4 days using digital calipers and tumor size calculated as the product of two perpendicular diameters.

For adoptive T cell therapy, NP68-B16 tumor bearing mice were sub-lethally irradiated with 597cGy total body irradiation (TBI) on day 6 following tumor cell injections. On day 7, mice were injected subcutaneously with 100 μg NP68 peptide in incomplete Freund's adjuvant (IFA; final volume of 200 μl) into the right flank prior to injection of T cells isolated from female donor mice into the tail vein. T cells and peptide were injected at 14.00–16.00 GMT and mice returned to their home cage. The checkpoint inhibitor, InVivoPlus anti-mouse PD-1, clone RMP1-14 (2BSCIENTIFIC), was injected on Day 10, 13, and 16 and InVivoMab anti-mouse CTLA-4 (CD152), clone 9H10, on Day 9, 12, and 15 (100 μg per injection). Other groups were treated with InVivoMAb rat IgG2a Isotype control (BE0089; 2BSCIENTIFIC) on the same days, times and doses as anti-PD-1 and InVivo MAb polyclonal Syrian Hamster IgG (BE087, 2BSCIENTIFIC) on the same days, times and doses as anti-CTLA-4. Antibodies were administered at 10.00–12.00 or 14.00–16.00 GMT and mice returned to their home cage.

To study the effect of regulatory T cell depletion, non-irradiated, NP68-B16 tumor-bearing FoxP3^DTR^ mice were injected intraperitoneally with 2.5 or 5 μg/kg diphtheria toxin (DT) 3 times a week or 100 or 200 mg/kg cyclophosphamide (CY) in 10% DMSO in PBS once a week starting at day 7 following tumor inoculation. A control group of tumor-bearing FoxP3^DTR^ mice received an equal volume of 10% DMSO in PBS once a week. Treatments were administered at 10.00–12.00 or 14.00–16.00 GMT and mice returned to their home cage.

At the end of the experiment, blood was collected from each mouse by cardiac puncture, serum prepared and stored frozen at −80°C. Tumors were excised, and either fixed in 10% neutral buffered formalin (NBF) and embedded in paraffin wax, or snap-frozen in OCT without fixative, or, in some cases, large tumors were bisected and treated with both preservation methods. Tumor growth rates were calculated as described previously (18). Briefly, tumor growth rate (k, days^−1^) was determined using a statistical software package Prism 5 (GraphPad) with the following equation for exponential growth: Y = Y0 × exp(k × X). Tumor diameter (X, mm) was measured every 3–4 days using calipers. On average, measurements were taken for 23 days for both untreated mice and F5 T cell treated mice. On average 5–7 measurements were used to calculate tumor growth rate.

### Disseminated Tumor Model

Adult male B6 mice were exposed to 597 cGy TBI on day 1 and randomly assigned to treatment groups of 8 mice per group for each experiment. For the therapeutic model, 5 × 10^6^ NP68-B16 tumor cells were injected intravenously on day 2. On day 4, 3.4 × 10^4^ T cells isolated from male donor mice were injected intravenously, followed by subcutaneous administration of NP68 peptide in IFA (**Figure 3A**). For the prophylactic model, timings of the tumor and T cell injections were reversed (**Figure 3A**). Mice were monitored until day 13 or 14 and then sacrificed. The lungs were excised, weighed, fixed in 10% NBF and embedded in paraffin wax. Five micrometer sections were stained with haematoxylin and eosin and tumor nodules analyzed by microscopy. In some studies, Thy 1.1 mice were injected with NP68-B16 tumor cells and the number and activation status of donor F5 T cells (Thy1.2^+^ CD8^+^) in the lungs at end stage (day 14) were determined by flow cytometry following tissue disaggregation.

### L-Selectin Dependent T Cell Homing

A short-term homing assay in which the recruitment of L-selectin expressing, and L-selectin deficient T cells are compared directly in individual mice was used as described previously (11). Briefly, Thy1.1 mice were subcutaneously injected with 1.5 × 10^6^ NP68-B16 tumor cells and tumors grown for 7–14 days. Thy1.1 mice served as non-tumor bearing mice. Tumor bearing mice and non-tumor bearing mice received 597cGy TBI. CD8^+^ T cells isolated from naïve F5LΔP and F5LselKO (both Thy1.2) were mixed 1:1 (numbers determined by haemocytometer, and confirmed by flow cytometry using Cytocount beads) and a total of 6–10 × 10^6^ T cells injected intravenously into mice at 14.00–16.00 GMT on the day following irradiation. All mice received 100 μg NP68-IFA s.c. prior to T cells. The next morning (10.00–11.00 GMT; 18 h following T cell injections), mice were culled and tissues including blood, spleen, tumor-draining and peptide-draining lymph nodes, non-draining lymph nodes and tumor were collected. Tumors were disaggregated using gentle MACS (Miltenyi). Lymphoprep was used to isolate immune cells from blood and tumor by centrifugation. Following isolation, cells were stained for CD8, Thy1.2, and L-selectin, analyzed by flow cytometry and L-selectin expression on donor CD8^+^, Thy1.2 cells determined. Cell numbers were determined using Cytocount beads (Dako).

### Longitudinal Tracking of Adoptively Transferred T Cells Using Micro PET/CT Imaging

^89^Zr was produced via proton bombardment of an Yttrium target *via* an adaptation of the methods of Walther et al. (24) and Dabkowski et al. (25). Briefly, a disk of natural abundance ^89^Y foil (300 μM thick, Goodfellow) in a custom made aluminum holder was loaded into a COSTIS Solid Target System (STS) fitted to an IBA Cyclone (18/9) cyclotron equipped with a 400 μM thick niobium beam degrader. The disk was irradiated for 4 h with a beam energy of 40 μA. The irradiated disk is left in the cyclotron for 12 h to allow any short lived ^89M^Zr to decay to ^89^Zr before removal for purification (activity 1.5–2GBq). The disk was dissolved in 2 M HCl with stirring and heat and the ^89^Zr was isolated by flowing over a hydroxymate functionalized ion exchange resin column (prepared in house freshly for each separation). The column was rinsed with 2 M HCl and water to remove ^89^Y before the ^89^Zr was liberated with 1 M oxalic acid in 3 × 1 ml fractions. The most concentrated fraction contained 800–1000MBq.

^89^Zr Oxine for cell labeling was prepared via an adaptation of the methods of Ferris et al. (26). Freshly prepared ^89^Zr Oxalate (200 μl, ~150–200 MBq) was adjusted to pH 7.0 with 0.5 M Na_2_CO_3_ (~270–390 μl) and diluted to 2 ml with distilled water in a 15 ml centrifuge tube. To this was added 2 ml of oxine solution in chloroform (1 mg/ml) and the resultant biphasic mixture was shaken at room temperature (RT) at 1,000 rpm for 1 h. The mixture was then allowed to settle and the lower chloroform layer was removed and the activity measured by dose calibrator (typically 1–20MBq). A further 2 ml of oxine chloroform solution was added to the remaining aqueous phase and the mixture was shaken overnight (1,000 rpm, RT). The resultant mixture was allowed to settle and the chloroform layer removed and the activity measured (typically 100–150 MBq). The chloroform was removed by heating and gentle stream of air until a dry dusty yellow crust remained in the vial. This was redissolved in DMSO (20 μl) and then made up to 2 ml with PBS for cell labelling.

CD8^+^ T cells were isolated from spleens and lymph nodes of naïve F5B6 and F5LΔP mice, resuspended to 13–66 × 10^6^/ml in PBS and incubated with ^89^Zr oxine (26–65 MBq) with shaking at 550 rpm for 30 min at RT. Unincorporated ^89^Zr oxine was removed by repeated washing in 10 ml PBS, cells collected by centrifugation (350 g, 5 min, RT) and supernatant checked for removal of free ^89^Zr. Uptake of ^89^Zr oxine by CD8^+^ T cells was 18–20% efficient, with labelled cell yields of between 30 and 50% and viability >90%. 8–17 × 10^6^ T cells labeled with 0.7–1.63 MBq ^89^Zr were resuspended in 200 μl PBS for injection into sub-lethally irradiated, tumor-bearing mice. One hundred microgram NP68-IFA was injected s.c. prior to the T-cell injection. Mice were anesthetized with isofluorothane (5% in O_2_ gas 1–2 l/min) and ^89^Zr labeled T cells injected intravenously. Mice were then injected with iopamidol contrast agent (Niopam 300, Bracco100 μl/mouse ip) and mice were scanned in a Mediso PET/CT Preclinical Imaging System (nanoScan122S PET CT Mediso). Respiration was monitored with a pressure pad connected to differential pressure transducers for low-range pressure monitoring during the entire PET-CT examination and anesthesia was maintained through the nose cone of the bed (1~2% isoflurane in O_2_ gas 1–2 l/min). PET emission data were collected for 60 min. Spatial resolution of PET measurements was 0.4 mm and energy lower/upper limit was 400/600. The CT scan parameters were set as follow: tube voltage was 50 kVp, tube current was 1 mA, exposure time 300 ms and maximum number of projections was 400. Reconstructed resolution of CT was 0.25 mm. PET-CT scans were repeated at 20, 44, 68, 140, 188, 212, and 235 h following T cell injections. Whole body radioactivity was independently measured immediately after scanning by placing the anesthetized mouse in a Capintec CRC-25 dose calibrator on calibrator setting 465.

PET/CT scans were analyzed using Vivo Quant software (inviCRO). Regions of interest were drawn over tumor, lung and skeleton for each mouse. Total radioactivity in each organ was calculated and expressed as percentage of total measured radioactivity in the mouse at each timepoint.

### Flow Cytometry

Cells were stained with fixable LIVE/DEAD™ Aqua dye as per the manufacturer's protocol (Invitrogen), treated with 50 nM dasatinib (Sigma) to prevent TCR downregulation, then stained with PE-conjugated NP68 tetramer at 37°C, followed by surface antibodies at 4°C as follows: anti-TCRVβ11-FITC, anti-CD8-PerCPCy5.5, anti-CD27-BV421, anti-CD44-APCCy7, anti-CD62L-PECy7, and anti-CD69-APC (all BioLegend). Cells were fixed with 10% formalin and data collected using a BD FACSCanto II within 4 days of staining. Between fixing and data collection, cells were stored in PBS 2% FCS at 4°C in the dark. Voltages and compensation were set using OneComp eBeads (eBioscience) and Arc-reactive beads (Invitrogen) and the FACSDiva automatic compensation function. CytoCount beads were used to quantify cell numbers, according to the manufacturer's instructions (Dako). Figures were prepared using FlowJo software (TreeStar Inc.).

### Immunohistochemistry

Formalin-fixed paraffin-embedded tumor tissue was cut into 5 μm sections and rehydrated. Antigen retrieval was performed using Tris-EDTA buffer (10 mM Tris, 1 mM EDTA, pH9). Sections were blocked with 0.5% H_2_O_2_ in methanol, rinsed in PBS, blocked with horse serum (Vector Laboratories) and stained overnight with rabbit polyclonal anti-CD3 (A0452, Dako) or rabbit polclonal anti-CD8 (BS-0648R, Biosciences). After staining with horse anti-rabbit-HRP (Vector Laboratories), sections were developed for 90–120 s with SG detection solution (Vector Laboratories). Slides were mounted using DPX (Fisher Chemical). Sections were photographed at 20 × magnification using an EVOS XL Core Microscope (Thermo Fisher Scientific).

### Quantification of CD3 and CD8 Staining

The whole tumor area was photographed at 20 × magnification. Five sequentially numbered fields of view (FOV) were analyzed per tumor. Where 5–6 FOV were taken, images 1–5 were analyzed; for 7–8 FOV, images 1, 3–5, 7 were analyzed; for 9 FOV, images 1, 3, 5, 7, and 9 were analyzed. Images were analyzed using the Fiji version of ImageJ. The scale was set to 3.4 pixels = 1 μm (based on image scale bar). Non-specific staining was subtracted using Background Correction, and the images were separated into FastRed, FastBlue, and DAB using Color Deconvolution. The FastBlue image was used to analyse CD3 and CD8. Minimum image threshold was set at 87, and maximum image threshold was set at 173–210. Percent area was analyzed and plotted. In cases where the tumor section did not cover the whole FOV, the blank area was subtracted prior to analysis. Four to twelve tumors were analyzed per treatment group.

### Soluble L-Selectin

Serum levels of L-selectin in tumor-bearing mice were quantified using a mouse L-Selectin/CD62L DuoSet ELISA (R&D Systems) and optical densities measured on a CLARIO microplate reader (430-0841).

### Statistics and Figures

Statistical analyses were performed in Prism 7 (GraphPad Software Inc.) as detailed in figure legends. Figures were prepared using FlowJo software (Treestar Inc.), Prism 7 (GraphPad Software Inc.), MS Powerpoint, and Adobe Illustrator.

## Results

### Solid Tumor Model to Study T Cell-Dependent Tumor Immunotherapy

**Figure 1:**
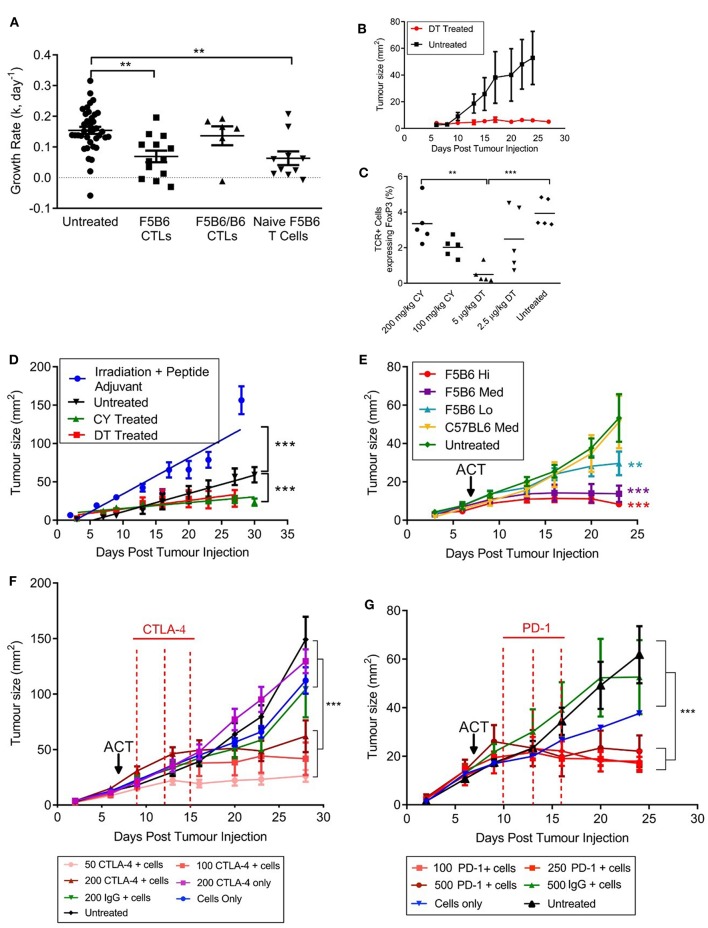
An immunogenic solid tumor model in mice. **(A)** B16 melanoma cells expressing the NP68 antigen were transplanted subcutaneously into immunocompetent mice. Seven days after tumor transfer, mice were treated intravenously with either naïve CD8^+^ T cells carrying the F5 TCR, or CD8^+^ T cells activated *in vitro* with NP68 peptide (CTLs). F5B6 cells are homozygous and F5B6/B6 cells are hemizygous for the F5 TCR. Growth rates of tumors over time were measured in two dimensions and a “k” value was calculated. Data from more than one experiment. Scatter plots show individual mice and bars show means and SEM. (Untreated, *n* = 41, F5B6 CTLs, *n* = 14; F5B6/B6 CTLs, *n* = 6; naïve F5B6 T cells, *n* = 10). **(B)** NP68-B16 cells were transplanted into FoxP3DTR mice, and 7 days following tumor transfer, half the mice were treated with 5 μg/kg DT diphtheria toxin (DT) every 3 days for 2 weeks, *n* = 6. **(C)** NP68-B16 cells were transplanted into FoxP3DTR mice, and 7 days following tumor transfer mice were treated with either 5 μg/kg DT, 2.5 μg/kg DT, 200 mg/kg cyclophosphamide (CY) or 100 mg/kg cyclophosphamide. After 7 days of treatment, peripheral blood was sampled from the tail vein, and the percentage of cells positive for TCR and FoxP3 was enumerated by flow cytometry. Scatter plots show individual mice and bars show means. **(D)** Tumor growth rates in mice receiving different host conditioning regimes. Mice were either untreated or conditioned 7 days following tumor transfer by sublethal irradiation and peptide adjuvant (C57BL/6 hosts), CY or DT treatment (FoxP3DTR hosts), *n* = 10–14. Data from more than one experiment. Differences in tumor growth rate were calculated by linear regression and significances indicate differences in slope. ***P ≤ 0.001. **(E)** C57BL/6 mice with NP68-B16 tumors established for 7 days were treated with sublethal irradiation (597cGy) and peptide adjuvant together with naïve CD8^+^ T cells carrying the F5 TCR or polyclonal T cells (C57BL6). T cell doses were 5 × 10^4^ = Lo; 2.25 × 10^5^ = Med; 5 × 10^5^ = High; *n* = 7–8. **(F)** C57BL/6 mice with NP68-B16 tumors established for 7 days were treated with sublethal irradiation (597cGy) and peptide adjuvant together with 2.25 × 10^5^ naïve CD8^+^ T cells carrying the F5 TCR in addition to treatment with anti-CTLA-4 antibody on days 9, 12, and 15 post tumor transfer, *n* = 8. **(G)** C57BL/6 mice with NP68-B16 tumors established for 7 days were treated with sublethal irradiation (597cGy) and peptide adjuvant together with 5 × 10^4^ naïve CD8^+^ T cells carrying the F5 TCR in addition to treatment with anti-PD-1 antibody at 10, 13, and 16 days post tumor transfer, *n* = 5–9. **(A,C)** Significance was calculated using one-way ANOVA with Tukey's multiple comparisons test. ***P ≤ 0.001, **P < 0.01. **(D–G)** Differences in tumor growth rate were calculated by linear regression and significances indicate differences in slope compared to untreated groups. **P ≤ 0.01, ***P ≤ 0.001.

To measure T cell responses in tumor-bearing mice we used a modified B16F10 cell line expressing the H2D^b^-restricted internal influenza nucleoprotein epitope NP68 (NP68-B16) as a surrogate tumor antigen (22). The lowest tumor cell dose that would consistently result in tumor growth in host animals was 5 × 10^5^ cells per animal and this dose was used throughout this paper. We used CD8^+^ T cells transgenic for the F5 TCR, which is specific for NP68 (20), as a source of tumor-specific T cells. Initial studies involved adoptive transfer of *in-vitro* activated CTLs expressing different levels of F5 TCR. CTLs hemizygous for F5 (F5B6/B6; Table 1) were not able to control the growth of NP68-B16 tumors (Figure 1A). However, F5B6 CTLs expressing increased levels of F5 TCR were able to control tumor growth (Figure 1A). We have previously shown that CTLs hemizygous for F5 control pulmonary influenza virus infections (11); the increased level of TCR required to control tumor growth most likely reflects the necessity to overcome tumor-induced immunosuppression. When we compared the ability of CD8^+^ T cells from naïve mice to CTLs, T cells from naïve mice were considerably more efficacious, with a dose of only 5 × 10^5^ T cells having the equivalent effect to 2 × 10^7^ *in vitro*-stimulated CTLs (Figure 1A). All subsequent experiments were conducted with CD8^+^ T cells freshly isolated from naïve mice, unless detailed otherwise.

To dissect the role of the host's immune system in this model, we transplanted the NP68-B16 tumor cells into FoxP3^DTR^ mice. These mice express the diphtheria toxin receptor on cells positive for the transcription factor FoxP3, which is expressed by regulatory T cells (Tregs). Once the tumors had been allowed to grow for 7 days, tumor-bearing mice were treated with 5 μg/kg diphtheria toxin (DT), to deplete Tregs (Figure 1B). This treatment completely halted tumor growth, indicating a very strong role for Tregs in restricting the growth of NP68-B16 melanomas. This also demonstrated that, once relieved of suppression by Tregs, the growth of solid tumors could be controlled by host T cells. The chemotherapeutic agent cyclophosphamide (CY) is thought to preferentially kill Tregs at low doses, due to their rapid division (27). Comparison of the prevalence of circulating Tregs in tumor-bearing hosts following either DT or CY treatment showed that 100 mg/kg CY affords a similar level of Treg depletion as DT treatment in FoxP3^DTR^ mice (Figure 1C). Furthermore, treatment with 100 mg/kg CY gave the same level of tumor growth control as DT treatment (Figure 1D). In contrast, treating tumor-bearing hosts receiving a non-lethal dose of radiation, together with a bolus of NP68 peptide in incomplete Freund's adjuvant (peptide-IFA) led to an increase in tumor growth (Figure 1D). Increased growth of NP68-B16 tumors following sub-lethal irradiation could reflect a loss of endogenous T cells which partially control tumor growth or a response to locally-induced inflammation which is known to promote tumor growth (28). Non-lethal doses of irradiation are commonly used to create niche space prior to adoptive cell transfer (ACT), and this pre-conditioning regime is employed throughout this paper. It is important to note that despite the clinical use of radiation for cancer treatment, in this model system, irradiation alone does not control tumor growth, and, in fact, accelerates growth. The final aspect of the model to establish is the therapeutic dose of T cells required to control tumor growth. In Figure 1E, we demonstrate that differing levels of tumor growth control can be produced by just 5-fold differences in F5 CD8^+^ T cell dose whereas equivalent doses of polyclonal CD8^+^ T cells do not control tumor growth.

We have demonstrated that the growth of NP68-B16 melanoma is controlled by the host immune system, including the use of Tregs to escape immune cell attack. However, in fully immunocompetent (non-irradiated) tumor bearing mice, the host immune response to NP68-B16 melanoma is not sufficient to control tumor growth (Figure 1D). Given this, we would expect checkpoint inhibitors to improve the control of these tumors, through augmentation of the host immune response. However, when tumor bearing mice are treated with anti-CTLA-4 monoclonal antibodies, mimicking the clinical ipilimumab therapy, improved control of tumor growth is only seen in conjunction with adoptive transfer of F5 T cells (Figure 1F). This demonstrates that although there is evidence in Figure 1D for a modest host immune response to the tumors, it remains insufficient for tumor control, even in the presence of checkpoint inhibition. This is in line with previously published observations of the poor immunogenicity of the unmodified B16 melanoma cell line (29). However, treatment with even a very low dose of anti-CTLA-4 acts synergistically with low dose ACT to produce striking control of tumor growth, even where ACT alone was not effective. The same powerful synergy is seen with anti-PD-1 monoclonal antibody treatment (Figure 1G).

### L-Selectin Enhanced T Cells in Adoptive Cell Therapy for Solid Tumors

**Figure 2:**
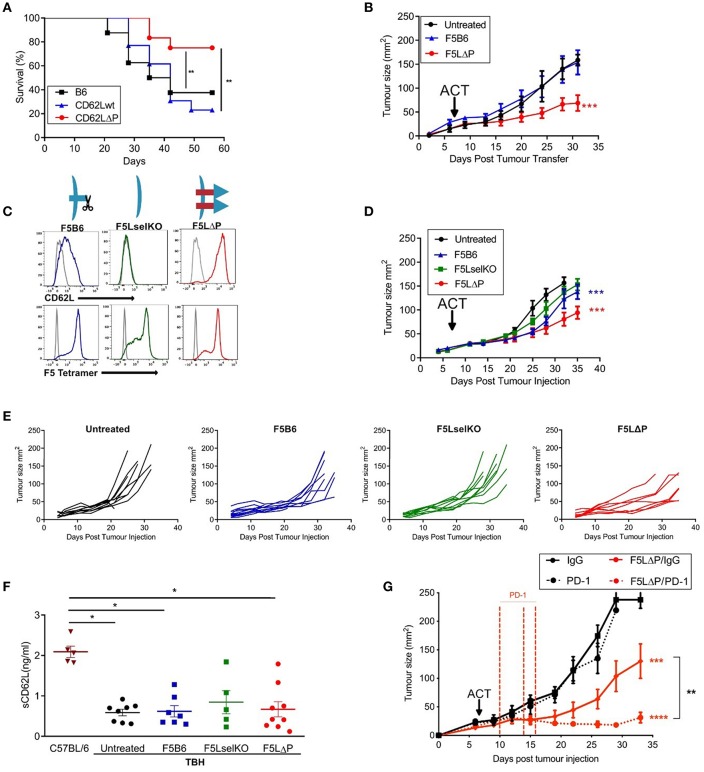
L-selectin enhanced T cells expressing gain-of-function L-selectin exhibit improved control of solid tumor growth. **(A)** B16F10 melanomas were grown subcutaneously in unmanipulated C57BL/6 (B6) mice or transgenic mice expressing either wildtype (CD62Lwt) or mutant (CD62LΔP) L-selectin and tumor growth measured in the absence of host conditioning. Data represent the survival of mice with tumors ≥ 200 mm^3^. *n* = 11–13 mice per group. **(B)** C57BL/6 mice with NP68-B16 tumors established for 7 days were treated with sublethal irradiation (597cGy) and peptide adjuvant together with 5 × 10^5^ naïve CD8^+^ T cells carrying the F5 TCR and either endogenous WT (F5B6) or gain-of-function transgenic L-selectin/CD62L (F5LΔP), *n* = 7–8. The untreated control group for this experiment has been published previously (22). The experiment was a multi-arm study, with a shared control group, for the purposes of Replacement, Refinement and Reduction of Animals in Research in the UK (3Rs). The F5 T cell-treated groups have not been published previously. **(C)** Flow cytometric analysis of CD8^+^ T cells prior to adoptive transfer, indicating levels of CD62L and transgenic F5 TCR. Gray histograms are background staining. Graphics show L-selectin expression and shedding in different T cell populations. **(D)** Effect of the F5 T cell populations shown in **(C)** on NP68-B16 tumors established for 7 days and conditioned with sublethal irradiation and peptide adjuvant, *n* = 10. **(E)** Tumor growth curves for individual animals in experiment **(D)**. **(F)** Soluble L-selectin in naïve C57BL/6 mice and tumor-bearing hosts (TBH) without (untreated) and with F5 T cell therapy. Symbols are data from individual mice. Results are mean + SEM. Significance tested using one-way ANOVA and Tukey's multiple comparison test. *P < 0.05. **(G)** Synergy between checkpoint blockade inhibition and L-selectin expressing T cells. Effect of F5LΔP T cells with and without anti-PD1 therapy (*n* = 8). Differences in tumor growth rate compared to untreated groups and between treatment groups were calculated by linear regression. *P ≤ 0.05, **P ≤ 0.01, ***P ≤ 0.001.

Subcutaneous B16F10 melanomas show accelerated growth in L-selectin knockout mice (30) but which L-selectin expressing leucocyte population controls B16F10 melanoma growth was not determined. To study the role of L-selectin on T cells, we generated gain-of-function L-selectin transgenic mice in which T cells express either wildtype (CD62Lwt) or a shedding-resistant form of L-selectin (CD62LΔP) expressed under a heterologous promoter which we have shown to abrogate cytokine induced transcriptional silencing in virus-infected mice (11, 13, 17). When parental B16F10 cells are transplanted into CD62Lwt mice, tumor growth was not significantly different than in the background C57BL/6 (B6) strain (Figure 2A), indicating that abrogating gene silencing of L-selectin on its own does not boost anti-tumor immunity over and above that seen in wildtype B6 mice expressing endogenous L-selectin. When parental B16F10 cells are transplanted into CD62LΔP mice, tumor growth is significantly slower than in either B6 or CD62Lwt mouse strains with survival of 80% in CD62LΔP mice compared with <40% in B6 and CD62Lwt mice (Figure 2A). These data demonstrate that abrogating ectodomain shedding as well as transcriptional silencing of L-selectin boosts T cell-dependent tumor immunity. Interestingly, this finding suggests that abrogation of shedding, rather than gene silencing, plays a dominant in promoting T cell dependent immune responses even against poorly immunogenic unmodified B16 melanoma cell line (29). CD62LΔP mice do not express endogenous L-selectin and expression of transgenic L-selectin is restricted to T cells (16). Together these data indicate that L-selectin expressed by T cells, and not by other leucocytes, regulates the growth of B16F10 melanomas. These data strongly support our hypothesis that L-selectin enhancement by maintaining expression on activated T cells improves T cell dependent immunity to tumors.

To determine whether this effect was due to the action of enhanced expression of L-selectin on antigen-specific T cells, CD8^+^ T cells from transgenic mice homozygous for both the F5 TCR and CD62LΔP (F5LΔP) were transferred into C57BL/6 mice with established NP68-B16 melanomas (Figure 2B). Expression levels of L-selectin and the transgenic F5 TCR were confirmed by flow cytometric analysis of transferred T cells (Figure 2C). Whilst CD8^+^ T cells expressing endogenous, wildtype L-selectin (F5B6) were unable to control tumor growth, L-selectin enhanced F5LΔP T cells produced a significant reduction in the rate of tumor growth (Figure 2B). To expand upon this, we repeated this study with F5 T cells that were either knockout or wildtype for L-selectin, and compared them to F5LΔP T cells (Figure 2D). As before, tumors were established for 7 days before ACT, and, as would be expected from the findings of Gattinoni et al. (14), cells that are knockout for L-selectin (F5LselKO) are unable to control tumor growth (Figure 2D). In contrast, F5LΔP T cells offer significantly improved control over F5B6 cells (Figure 2D). The improved control of tumor growth may be seen in more detail by viewing the individual tumor growth curves for each mouse, displayed as average curves in Figure 2E.

Ectodomain shedding of L-selectin on T cells and other leucocytes releases soluble L-selectin into the bloodstream which can compete for ligands on blood vessels and restrict L-selectin dependent leucocyte recruitment (31). Blood levels of soluble L-selectin in F5LΔP and LΔP mice are <5% of those in matched control mice expressing wildtype L-selectin due to lack of homeostatic and activation induced shedding (16, 19). To determine if the increased efficacy of F5LΔP T cells is dependent on altered blood levels of soluble L-selectin, soluble L-selectin in peripheral blood was measured at the time of tumor harvest. As shown in Figure 2F, soluble L-selectin was detected in tumor-bearing mice in the complete absence of T cell transfers. Moreover, levels of soluble L-selectin did not change following transfer of F5LselKO T cells, F5B6 T cells, or F5LΔP T cells (Figure 2F) showing clearly that soluble L-selectin in tumor-bearing mice is not derived from injected donor T cells but, instead, is derived from host leucocytes. Interestingly, soluble L-selectin levels in tumor-bearing mice were 60% lower than in age- and sex-matched, naive C57BL/6 mice. Although not investigated further, this may be due to reconstitution of the immune system following sublethal irradiation. Together, these findings show that the increased efficacy of F5LΔP over F5B6 and F5LselKO T cells was not dependent on altered blood levels of soluble L-selectin in tumor-bearing mice, but instead depended on increased and maintained expression of L-selectin at the surface of F5LΔP T cells.

To assess the potential for L-selectin enhanced T cells to complement other immunotherapeutic strategies, we determined the efficacy of F5LΔP T cells in the presence and absence of PD-1 blockade. In Figures 2B,D, the benefit of maintained expression of L-selectin over wild type in the treatment of solid tumors is clearly shown. When F5LΔP T cells are combined with anti-PD-1 therapy, a striking synergistic effect is seen, with tumor growth being arrested following combinatorial treatment (Figure 2G), demonstrating the clear potential of combining F5LΔP T cells with checkpoint blockade inhibition.

### L-Selectin Enhanced T Cells in Adoptive Cell Therapy for Disseminated Tumors

**Figure 3:**
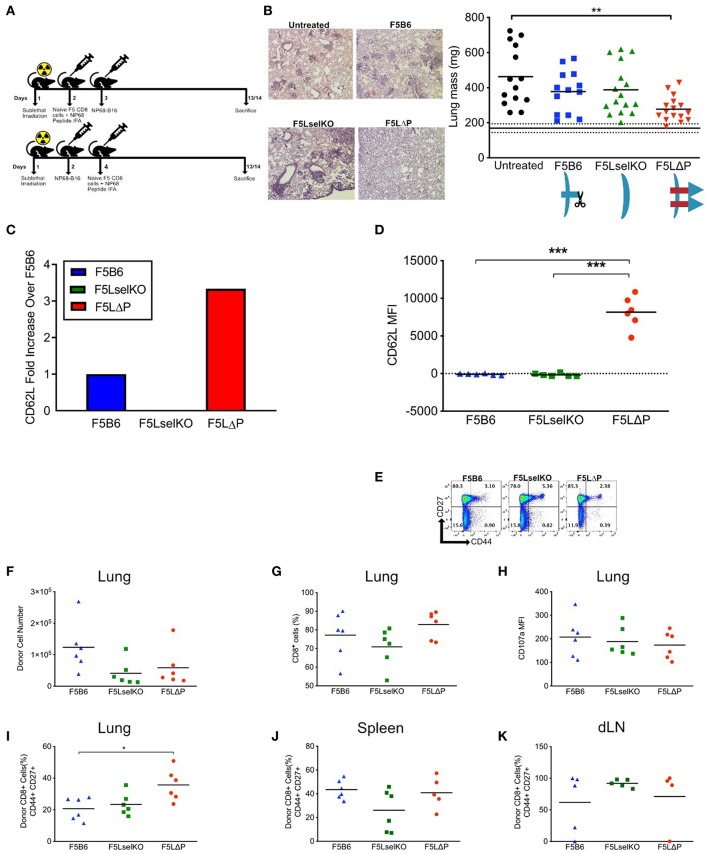
L-selectin enhanced T cells expressing gain-of-function L-selectin exhibit improved control of disseminated tumor growth. **(A)** Experimental protocol schematic. Both NP68-B16 tumor cells and naïve CD8^+^ cells were introduced via intravenous injection. **(B)** Tumor load measured by representage images of H&E stained lung sections and total lung mass. Data are pooled from more than one experiment. Symbols show individual mice and bars show means. Solid and dashed black lines show mean lung weight ± SD in age- and sex-matched naïve mice^1^. Graphic shows L-selectin expression and shedding in different T cell populations. **(C)** Fold increase in expression of CD62L on F5LΔP naïve CD8^+^ T cells compared to F5B6 and F5LselKO CD8^+^ T cells. **(D)** CD62L expression of donor T cells recovered from the lungs of tumor bearing hosts on day 14, measured by mean fluorescence intensity. **(E)** CD44 and CD27 expression on donor CD8^+^ T cells prior to adoptive transfer. **(F,G)** Donor cells recovered from tumor-bearing lungs, by total number **(F)** and percentage CD8^+^ cells **(G)**. **(H)** Expression of CD107a on donor T cells recovered from tumor-bearing lungs, measured by mean fluorescence intensity. **(I)** Percentage of donor T cells recovered from tumor-bearing lungs that are positive for CD44 and CD27. **(J)** Percentage of donor T cells recovered from spleen that are positive for CD44 and CD27. **(K)** Percentage of donor T cells recovered from the lung-draining lymph nodes that are positive for CD44 and CD27. **(B,D,F–K)** Significance was calculated using one-way ANOVA with Tukey's multiple comparisons test. ***P ≤ 0.001, **P ≤ 0.01, *P ≤ 0.05.

To determine if L-selectin enhanced T cells confer protection against disseminated tumors, we used NP68-B16 tumor cells administered via the bloodstream which results in colonization of the lungs in the absence of a primary solid tumor. We tested the impact of enhanced L-selectin on the outcome of T cell therapies delivered either before or after intravenous injection of tumor cells. In a prophylactic model, F5 T cells (Thy1.2) expressing variable levels of L-selectin were injected intravenously into sub-lethally irradiated (Thy1.1) mice and mice vaccinated in the right flank with NP68 peptide in adjuvant. NP68-B16 tumor cells were injected intravenously the following day. In a therapeutic model, NP68-B16 tumor cells were intravenously injected into sub-lethally irradiated mice and allowed to colonize the lungs. Two days later, F5 T cells expressing variable levels of L-selectin were injected intravenously and mice vaccinated in the right flank with NP68 peptide in adjuvant (Figure 3A). In both models, mice were harvested 2 weeks after tumor administration, lungs excised, and tumor loads determined by total lung weight and confirmed by histology. F5 T cells deficient in L-selectin or expressing wildtype L-selectin did not affect tumor loads in either the prophylactic or therapeutic models when compared with tumor-bearing mice that did not receive T cells (Figure 3B). However, F5LΔP T cells significantly reduced tumor load in both models and average lung weights reduced from 463 mg in untreated mice to 277 mg following F5LΔP T cell therapy which approached that of age- and sex-matched non-tumor bearing mice at 169 mg (Figure 3B). The improved efficacy of F5LΔP T cells was also evident by the absence of tumor nodules and normal appearance of the lungs (Figure 3B). Prior to injection, the mean level of L-selectin on F5LΔP T cells was 3-fold higher than on F5B6 T cells (Figure 3C), as reported previously (19). L-selectin expression was maintained on F5LΔP T cells but completely downregulated on F5B6 T cells within 2 weeks of transfer into tumor bearing mice (Figure 3D) demonstrating clearly the ability of the genetic mutant to resist ectodomain shedding as well as transcriptional silencing in tumor-bearing mice. The activation status of injected T cells was not affected by L-selectin expression and injected F5B6, F5LselKO, and F5 LΔP T cells comprised >94% CD44^lo^ naïve T cells (Figure 3E). The numbers, frequencies and extent of activation-induced degranulation (measured by CD107a mobilization) of donor derived F5LΔP T cells in the lungs after 14 days were not significantly different from either F5B6 or F5LselKO T cells (Figures 3F–H). The frequencies of CD44^+^CD27^+^ central memory T cells were higher in the F5LΔP population in the lungs, but not in the draining lymph nodes (LN) or spleen (Figures 3I–K). Therefore, maintained L-selectin appears to have a role in the activation of tumor-specific T cells, as indicated by CD44 and CD27 expression at the site of the tumor, but not in tumor-draining LN or spleen.

### Impact of L-Selectin on T Cell Homing in a Solid Tumor Model

**Figure 4:**
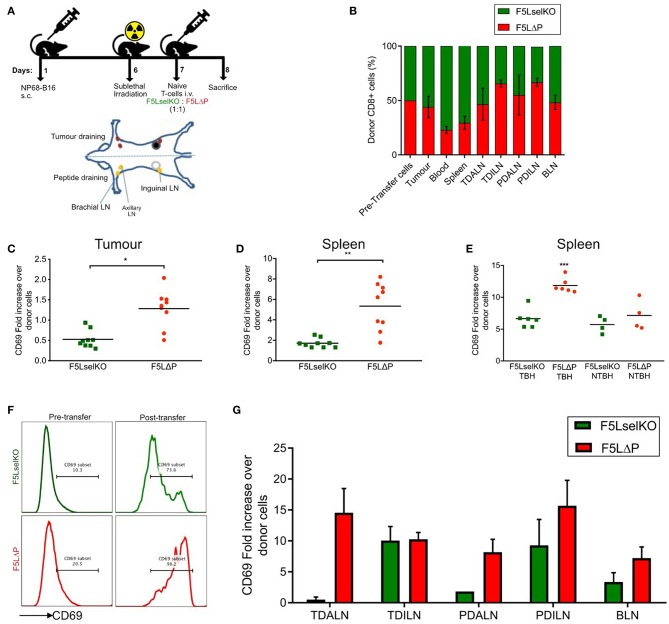
L-selectin dependent T cell homing and activation in tumor bearing mice. **(A)** Schematic of *in vivo* homing study and the location of lymph nodes in relation to tumor and peptide adjuvant injections. Diagram is of ventral view of mice showing various LNs. **(B)** Competitive homing of L-selectin sufficient (F5LΔP) and L-selectin deficient (F5LselKO) naïve T cells. T cells were allowed to home for 18 h before being recovered and analyzed by flow cytometry based on expression of F5 and L-selectin transgenes. Values are normalized to a total donor cell population of 100%. TDALN, tumor draining axillary lymph node; TDILN, tumor draining inguinal lymph node; PDALN, peptide draining axillary lymph node; PDILN, peptide draining inguinal lymph node; BLN, brachial lymph node. **(C–G)** Expression of activation marker CD69 on donor T cells recovered from tumor, spleen and LN calculated as fold increase over pre-transfer expression. Pre-transfer CD69 MFI values were 1,580 for F5LselKO cells and 1,664 for F5LΔP cells. **(C)** CD69 expression levels on donor T cells recovered from tumors of mice 18 h after injection. **(D)** CD69 expression levels on donor T cells recovered from spleens of tumor-bearing hosts after 18 h. **(E)** Comparison of CD69 expression on donor cells recovered from the spleens of tumor-bearing hosts (TBH) and non-TBH after 18 h, in a competitive homing study similar to that described in **(A)**. **(F)** Representative plots showing CD69 expression on donor T cell pre-transfer to tumor bearing mice and following isolation from spleens of tumor-bearing mice (post-transfer). Percentages of CD69 positive donor T cells are shown. **(G)** Comparison of CD69 expression on donor cells recovered from the LN of tumor-bearing hosts after 18 h, in a competitive homing study similar to that described in **(A)**. **(B,G)** Bars show means ± SEM (*n* = 5). **(B–E,G)** Significance was calculated using two-way ANOVA and multiple *t*-test. ***P ≤ 0.001, **P ≤ 0.01, *P ≤ 0.05.

L-selectin is an obligate homing molecule on naïve T cells for recruitment from the bloodstream into subcutaneous (peripheral) lymph nodes via specialized high endothelial venule (HEV) blood vessels (32). Previous studies by Klebanoff et al. (15) showed that adoptive T cell therapy of L-selectin expressing CTLs did not have any anti-tumor effect in lymphotoxin-α deficient mice, which lack lymph nodes. It was concluded that the role of L-selectin in adoptive T cell therapy is to direct homing of adoptively transferred CTLs to LN, although T cell homing to lymph nodes was not studied. HEV-like blood vessels expressing peripheral node addressin (PNAd), a ligand for L-selectin, have been reported in B16F10 melanomas and shown to support L-selectin dependent recruitment of T cells (33). This raises the possibility that L-selectin promotes homing of adoptively transferred naïve T cells from the bloodstream into the tumor via PNAd expressing tumor blood vessels, where T cells could be activated to kill tumor cells, without the need for LN entry. To determine the role of L-selectin in T cell homing to LN and tumors in tumor-bearing mice, we used a competitive homing assay that we have previously employed to determine the role of L-selectin in recruitment of CTLs from the bloodstream into virus-infected lungs (11). F5LΔP and F5LselKO T cells (both Thy1.2) are mixed 1:1 and injected into Thy1.1 tumor-bearing mice. This assay avoids the use of cell tracker dyes which may affect T cell trafficking and, unlike cell tracker dyes, the Thy1 marker is not diluted through cell division. It also exploits the fact that L-selectin sufficient T cells (F5LΔP) can be distinguished from L-selectin deficient (F5 LselKO) T cells due to L-selectin expression which is maintained following T cell activation (11) and, as shown here, in tumor-bearing mice (Figure 3D). The use of F5LΔP T cells also prevents the loss of L-selectin from T cells after they have been recruited from the bloodstream into cancerous tissues. After 18 h, the ratio of F5LΔP:F5LselKO T cells in tumors, spleen, blood, tumor-draining and peptide-draining inguinal and axillary LN, and brachial LN were analyzed by flow cytometry (Figure 4A). In our experience, brachial LN are not involved in draining the tumor in this model, whereas both inguinal and axillary LN have been observed to be tumor draining (data not shown). The brachial LN are therefore included to represent uninvolved, peripheral LN which depend on L-selectin for naïve T cell recruitment from the bloodstream via HEV (32).

L-selectin sufficient and L-selectin deficient T cells were detected in approximately equal numbers in all peripheral LN analyzed (tumor-draining and peptide-draining inguinal and axillary as well as brachial LN) (Figure 4B). Although not significant, L-selectin expression conferred slightly improved homing to inguinal LN, but not to other peripheral LN (Figure 4B). These findings are in marked contrast to published data including own our studies in naïve and virus-infected mice where peripheral LN are highly enriched in L-selectin positive T cells (11, 16, 32). L-selectin sufficient and L-selectin deficient T cells were both found in the spleen (which lack HEV), as reported previously (32), however, a higher proportion of L-selectin-deficient cells was observed in the blood which fits with previous observations that these cells are unable to traffic from the bloodstream into tissues (32). In contrast to ^1^ expectations, F5 T cells were found associated with solid tumors 18 h following intravenous administration. Moreover, L-selectin did not improve early homing of transferred T cells to the tumor, with the ratio of F5LΔP:F5LselKO cells harvested from resected tumors remaining similar to the injected population. These results show clearly that the improved control of tumor growth by L-selectin enhanced T cells is not simply explained by increased homing either to LN or to the tumor within the first 18 h following ACT.

### Impact of L-Selectin on T Cell Activation in a Solid Tumor Model

The unexpected homing of L-selectin deficient T cells to peptide-draining and tumor-draining peripheral LN of tumor bearing mice, as well as to tumors, suggests that the lack of tumor growth control by F5LselKO T cells is not simply related to an inability to home to sites of tumor antigen-presentation in LN or tumors. In the absence of a clear role for L-selectin in T cell homing, we considered whether L-selectin expression controls T cell activation in lymphoid organs or inside tumors. The expression of early activation marker CD69 on donor cells on the day of transfer in the competitive homing assay was quantified by median fluorescence intensity (MFI). Fold increase in expression of CD69 was subsequently calculated based on the MFI of donor T cells following recovery from tissues at the end of the study, compared to CD69 expression on T cell populations prior to transfer. CD69 expression was upregulated on L-selectin sufficient (F5LΔP) T cells harvested from tumor tissues 18 h following transfer to tumor-bearing mice whereas CD69 on L-selectin deficient T cells (F5Lselko) was not increased (Figure 4C). CD69 expression was upregulated on both L-selectin sufficient and L-selectin deficient T cells harvested from spleen of tumor bearing mice, however it was significantly higher on F5LΔP T cells compared to F5LselKO T cells (Figure 4D). When L-selectin sufficient and deficient cells were harvested from the spleens of non-tumor bearing mice which had undergone sub-lethal irradiation and peptide vaccination, there was a uniform increase in CD69 expression in both F5LΔP and F5LselKO T cells. These data show that the conditioning regime of irradiation and peptide vaccination increases CD69 equally on injected F5LΔP and F5LselKO T cells but it does not contribute to L-selectin-dependent increases in CD69 expression. Rather, the higher levels of CD69 on L-selectin sufficient T cells in the spleens of tumor-bearing mice are a direct consequence of tumor load (Figure 4E). Representative plots showing the extent of CD69 upregulation on injected T cells in the spleens of tumor-bearing mice are shown in Figure 4F.

In contrast to the spleen, CD69 expression on F5LΔP T cells and F5LselKO T cells harvested from LN was variable and depended on LN location. In tumor-draining and peptide-draining axillary LN, CD69 was higher on L-selectin sufficient T cells whereas in tumor-draining and peptide-draining inguinal LN, CD69 was upregulated equally (Figure 4G). CD69 was upregulated on both L-selectin sufficient and L-selectin deficient T cells in brachial LN (Figure 4G). As brachial LN is not a site of tumor antigen presentation, this finding suggests a role for the host conditioning regime in upregulating CD69 on F5LΔP T cells and F5LselKO T cells as found in the spleens of non-tumor bearing mice (Figure 4E). Together, these findings suggest that L-selectin expression on adoptively transferred T cells does not promote homing to tumors or to LN. Instead, L-selectin expression promotes early T cell activation as measured by CD69 expression inside the tumor, spleen and involved LN.

### Longitudinal Tracking of ^89^Zr Labeled T Cells in Tumor Bearing Mice Using PET-CT

**Figure 5:**
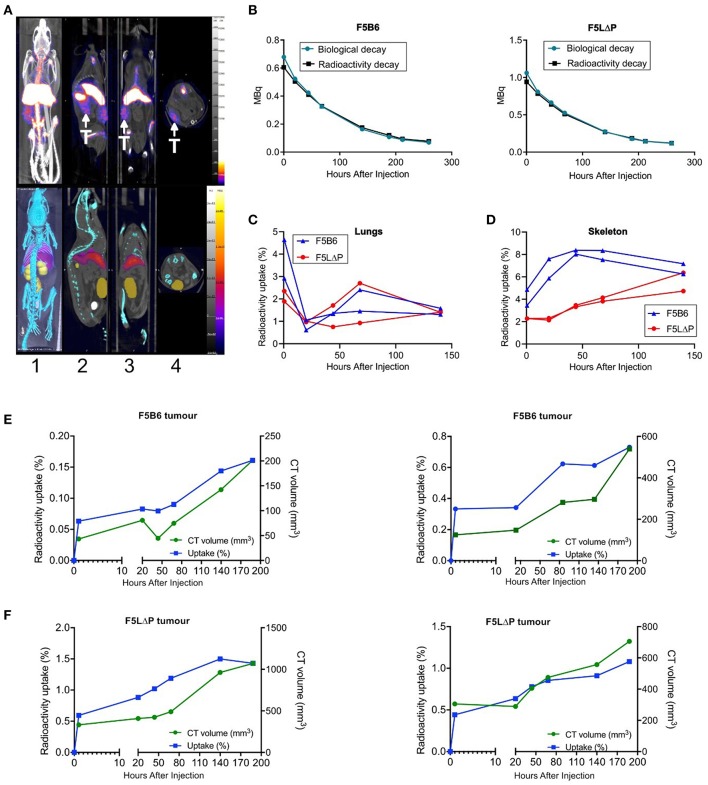
Longitudinal tracking of ^89^Zr labelled T cells in tumor bearing mice using PET-CT. **(A)** Representative PET-CT images of NP68-B16 tumor bearing B6 mice 5 days after intravenous injection of ^89^Zr labeled T cells. T, tumor. Image orientations: 1, Maximum intensity projection; 2, Sagittal; 3, Coronal; 4, Transverse. **(B)** Representative time-activity curves from dosimeter quantification of ^89^Zr in the whole mouse overlaid with predicted radioactive decay of ^89^Zr in recipients of F5B6 (left) and F5LΔP (right) T cells. **(C,D)** Time-activity curves from image-based quantification of ^89^Zr in the lungs **(C)** and skeletons **(D)** of 2 mice receiving F5B6 (blue) or F5LΔP (red) T cells expressed as percentage of total ^89^Zr in the mouse at each timepoint. The earliest time point in **(B–D)** is 1 h after injection. **(E,F)** Time-activity curves from image-based quantification of ^89^Zr in tumors expressed as percentage of total ^89^Zr in the mouse (blue line) overlaid with tumor size measured by CT (volume; green line). Graphs show overlaid curves from each of two mice receiving either F5B6 **(E)** or F5LΔP **(F)** T cells.

The localization of CD8^+^ T cells isolated from naïve mice to tumors within 18 h following intravenous injection was unexpected. It was predicted that T cells would require prior activation by peptide-MHC complexes on APC in lymphoid organs and subsequent differentiation to effector T cells before being able to enter tumors from the bloodstream. T cell activation takes longer than the 18 h timeframe of the homing study (11, 34). To study the kinetics of T cell recruitment into tumors in individual mice following adoptive T cell therapy, we labeled F5 CD8^+^ T cells *ex vivo* with ^89^Zr oxine, as described previously for human T cells (35, 36), and injected ^89^Zr-labeled T cells to tumor bearing mice following the normal conditioning protocol of sub-lethal irradiation and peptide vaccination. Mice were scanned within 1 h following injection of T cells, rescanned the following day and at 1–2 day intervals thereafter. ^89^Zr was detectable in tumors, lungs, skeleton, liver, and spleen within 1 h following injection and for the following 5 days, but was not detected in heart, kidney or bladder (Figure 5A and data not shown). To determine whether ^89^Zr remains associated with T cells following injection into mice or is released and excreted as waste, the predicted radioactive decay curve for total injected ^89^Zr was plotted alongside the actual amount of ^89^Zr detected in individual mice receiving either F5B6 or F5 LΔP T cells. The total amount of ^89^Zr detected in a single mouse completely overlaid the predicted radioactive decay curves for both types of T cell indicating that cell-free ^89^Zr was not excreted during the course of the study (Figures 5B,C). We exploited the known kinetics of temporary intravascular trapping and release of T cells from the lungs (37) over the first 24–48 h following intravenous injection to assess whether ^89^Zr remains associated with T cells. The fraction of total ^89^Zr in the lungs of mice receiving F5B6 T cells fell from 3 to 5% at 1 h to ~1% at 20 h after which it gradually increased over the following 5 days (Figure 5C). Lung associated ^89^Zr in F5LΔP recipients showed similar kinetics decreasing from 2% at 1 h to <1% at 20 h (Figure 5C) suggesting that ^89^Zr is T cell associated, at least for the first 20 h following injection. The fraction of total ^89^Zr in the skeletons of mice receiving F5B6 T cells was ~4% 1 h after injection, increased gradually and stabilized at 6–8% at 44 h after injection (Figure 5D). Skeletal localization was slightly lower in mice receiving F5LΔP T cells at 2% after 1 h and gradually increased to ~4% over the next 6 days (Figure 5D). Skeletal localization of ^89^Zr did not exceed 8% of total ^89^Zr, even after 140 h, which supports the hypothesis that the bulk of ^89^Zr is not being released as cell-free ^89^Zr during the course of the experiment which would be expected to localize to the skeleton (38). ^89^Zr was detectable in tumors within 1 h following intravenous injection of either F5B6 or F5LΔP T cells and ^89^Zr levels increased as tumors grew (Figures 5E,F). ^89^Zr in tumors fell below the level of detection after 188 h, which is before the effects of ACT are detectable. Although these data suggest that ^89^Zr remains T cell associated for 7 days, we cannot formally exclude localization of ^89^Zr in macrophages following death of T cells at time points beyond 20 h. These studies did not allow a direct comparison between F5B6 and F5LΔP T cells in the same mouse, however, they do show clearly that both T cell populations localize to tumors within 1 h of injection into the bloodstream. Taken together with the 18 h homing data, this shows that there is extremely rapid recruitment of T cells from the bloodstream and entry into solid tumors that is not dependent on L-selectin, followed by ongoing T cell accumulation over more than a week.

### End Stage Analysis of TILs and T Cell Proliferation

**Figure 6:**
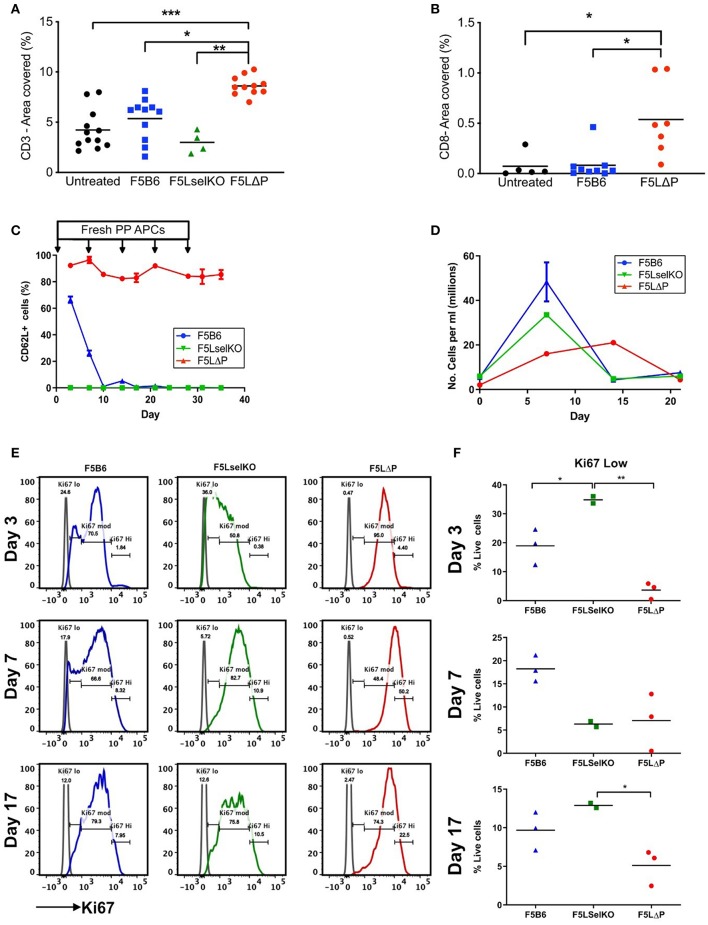
Impact of L-selectin on TILs and T cell proliferation *ex vivo*. **(A,B)** Subcutaneous tumors were fixed in formalin and embedded in paraffin. Five micrometer sections were stained for CD3 **(A)** and CD8 **(B)** and images analyzed using Fuji Image J software. Each symbol represents % area stained in tumors from single mice receiving no T cells (untreated), T cells expressing endogenous L-selectin (F5B6), no L-selectin (F5 LselKO), or enhanced L-selectin (F5LΔP). Data are pooled from more than one experiment conducted under identical conditions. Bars show means (Untreated, *n* = 5–12, F5B6, *n* = 9–11; F5LselKO, *n* = 4; F5LΔP, *n* = 7–10). **(C)** CD8^+^ T cells from F5B6, F5LΔP, and F5LselKO mice were stimulated *in vitro* on NP68-peptide pulsed APCs, with restimulation every 7 days. Every 3 days, a sample of cells was collected, and analyzed by flow cytometry for a range of markers, including CD62L, *n* = 2–3. **(D)** Total live cells were enumerated by haemocytometer at each timepoint. **(E)** The proliferation marker Ki67 was compared on the same cell populations at days 3, 7, and 17. **(F)** The frequency of Ki67^lo^ cells at each time point. **(A,B,F)** Significance was calculated using one-way ANOVA with Tukey's multiple comparisons test. ***P ≤ 0.001, **P ≤ 0.01, *P ≤ 0.05.

To determine if maintained L-selectin alters the number of tumor infiltrating lymphocytes (TILs), the total numbers of CD3^+^ and CD8^+^ T cells were enumerated in tumor sections from solid tumors harvested at the end of the study (this could represent either an individual mouse culled due to tumor size, or animals sacrificed as a group at the end of the entire study). Mice were pooled from more than one experiment where experimental conditions were the same. As shown in Figure 6A, host-derived CD3^+^ T cells were readily detectable in tumors of mice which did not receive T cells (untreated). The total numbers of CD3^+^ TILs (donor and host) were not significantly increased following transfer of either F5B6 or F5LselKO T cells. The total number of CD3^+^ TILs in the F5LΔP treatment groups was significantly increased by approximately 2-fold. Similarly, the total number of CD8^+^ TILs in F5LΔP treated mice was significantly higher than in either F5B6 treated or untreated mice which had similar numbers CD8^+^ TILs (Figure 6B). The classical ligand for L-selectin, PNAd, was not detectable on tumor blood vessels using MECA79 antibody, although MECA79 positive HEV were detected in peripheral LN of naïve mice using the same staining procedure used for tumors (data not shown).

These data show that the increased number of CD3^+^ TILs in F5LΔP recipients is not simply related to increased L-selectin dependent recruitment from PNAd expressing blood vessels inside the tumor either at the start of or during the course of ACT. This is supported by the disseminated tumor model, which does not rely on disordered tumor vasculature for T cell entry, but instead utilizes the established and extensive lung vasculature. No increase in injected T cells was observed in the lungs of tumor-bearing mice, despite improved tumor control by F5LΔP T cells. Instead, it could reflect increased retention due to higher CD69 expression and possibly increased frequency of central memory CD44^+^ CD27^+^ T cells at the tumor site which are known to be more efficacious in mouse models of ACT (15), as found in the disseminated tumor model (Figure 3I). We also considered whether the level of L-selectin on T cells determines other T cell properties that could contribute to the control of tumor growth. Cross-linking of L-selectin costimulates antigen-induced T cell proliferation (39) and controls important effector functions such as superoxide production (40), colony-stimulating factor 1 release (41) and lytic activity (42), all of which may contribute to tumor cell killing. Our previous studies of T cell dependent virus immunity have demonstrated that ectodomain shedding of L-selectin controls the clonal expansion of CD8^+^ T cells (43) so we started by analyzing the impact of L-selectin on T cell proliferation. In the mouse tumor model, F5 T cells are exposed to NP68 cognate peptide expressed on tumor cells and to exogenously injected or tumor derived peptide on APC in spleen and LN, as evidenced by CD69 upregulation at these sites (Figures 4E–G). To simulate continuous exposure of T cells to tumor antigen and measure T cell proliferation we exposed isolated T cells to peptide-pulsed APC *in vitro* and measured T cell proliferation and expression of the proliferation marker Ki67. Peptide stimulation was repeated every 7 days. As shown in Figure 6C, L-selectin expression is downregulated on F5B6 T cells over the first 7 days of stimulation and remains low following subsequent restimulations whereas F5LΔP cells maintain high levels of L-selectin throughout primary and subsequent re-stimulations. F5B6 undergo rapid clonal expansion during primary stimulation and T cell numbers peak after 7 days at 50 × 10^6^/ml and gradually decline following restimulation (Figure 6D). Proliferation of F5LselKO T cells followed similar kinetics as F5B6 cells showing early clonal expansion and subsequent contraction after 7 days. However, F5LΔP T cells showed a lag in proliferation which peaked later at 14 days. Moreover, the extent of clonal expansion of F5LΔP T cells was smaller resulting in a 10-fold increase in T cell numbers at day 14, compared to F5B6 and F5LselKO T cells which increased by ~20-fold by day 7. Analysis of Ki67 expression revealed differential patterns of expression in all three T cell populations. Expression levels were categorized as low, medium or high (Figure 6E) and showed different frequencies at each level between F5B6, F5LΔP, and F5LselKO T cells. Statistically significant differences in the frequencies of Ki67 low cells were seen at day 3 and day 17 but not at later times (Figure 6F). The decrease in the number of F5LΔP cells that are Ki67 low suggests that, despite the slower and overall lower level of proliferation, expression of the proliferation marker Ki67 was higher in F5LΔP cells. Whether this assay replicates the stimulation and expansion of F5LΔP T cells in tumor bearing mice is not known. However, it demonstrates that L-selectin enhanced T cells behave differently to cells expressing either wildtype endogenous L-selectin or no L-selectin in response to immune stimulation in a model system where cell trafficking is not relevant.

## Discussion

L-selectin (also known as CD62L) is an important homing molecule on the surface of T cells for their recruitment from the bloodstream into lymph nodes where T cells first become activated by peptide-MHC complexes on dendritic cells. L-selectin (CD62L), is commonly used as a marker of T cell activation, due to the well-documented observation that expression of L-selectin is downregulated when T cells are activated to CTL status. In mouse models of ACT, studies have shown that even when T cells are matched for activation status, L-selectin expressing CTLs confer an advantage over L-selectin knockout CTLs in controlling tumor growth (14). Based on these findings, we investigated the hypothesis that CTLs expressing a “gain-of-function” form of L-selectin that is never downregulated, even following T cell receptor engagement (LΔP; L-selectin enhanced), are better able to control the growth of solid cancers than wild type T cells which downregulate L-selectin expression. We report here that L-selectin enhanced T cells show improved control of tumor growth. Previous studies have linked L-selectin to T cell function due to the differential homing of L-selectin expressing naïve/central memory T cells to lymph nodes and L-selectin negative effector/effector memory T cells to non-lymphoid/ inflamed tissues. In this study, we show clearly that the effect of L-selectin on tumor growth is unrelated to L-selectin dependent homing but instead correlates with early activation of therapeutic T cells inside tumors.

In two different tumor models, T cells expressing LΔP-selectin were better able to control the growth of transplanted tumors than wild type T cells. These findings support the hypothesis of the study and show clearly that maintaining L-selectin expression is beneficial for adoptive T cell tumor therapy. L-selectin enhanced T cells controlled the growth of solid, vascularized subcutaneous tumors as well as disseminated tumors which had colonized the lungs showing that the effect of L-selectin enhanced T cells is not restricted by tumor location or tumor size at the start of therapy. Clinically, the most marked effects of cancer immunotherapy have been achieved through the use of checkpoint blockade inhibitors. In the solid tumor model, checkpoint blockade inhibitors did not control tumor growth in the absence of T cell transfers. However, anti-CTL-4 and anti-PD-1 monoclonal antibody therapy showed marked synergy with the transfer of T cells expressing wild type L-selectin resulting in arrest of growing tumors. This powerful synergy is also demonstrated by the combination of anti-PD-1 with F5LΔP CD8^+^ T cell transfer.

The obvious mechanism for control of tumor growth by F5 LΔP T cells would be improved trafficking into the tumor. Indeed, the motivation behind applying this molecule to ACT is based on our own findings of improved anti-viral immunity due to homing of LΔP T cells to virus-infected lungs (11). Increased numbers of CD3^+^ and CD8^+^ cells were identified in solid tumors in mice treated with F5LΔP cells compared to either wild type or L-selectin knockout T cells at experimental endpoints. However, increased TILs in solid tumors up to 21 days after transfer could be due to a range of scenarios, including improved homing, increased proliferation, increased retention or prolonged survival. To determine the kinetics of T cell recruitment from the bloodstream into solid tumors, we tracked ^89^Zr labeled T cells following transfer to tumor-bearing mice by PET/CT. Since therapeutic T cells were not pre-activated prior to transfer, we expected a lag between T cell injection and localization of CTLs inside tumors which would reflect activation in secondary lymphoid organs of tumor-bearing mice. However, tumor-specific T cells were detected inside tumors within 1 h of injection into the bloodstream. ^89^Zr label steadily increased inside solid tumors over the subsequent 8 days of tumor growth which is before the therapy took effect. Wild type T cells and L-selectin enhanced T cells were tracked in the tumor at every time point including 1 h after transfer. Although direct comparison of different T cell populations in the same tumor-bearing mouse was not possible using this method, it did demonstrate that F5B6 (unmodified) T cells are able to enter the tumor, and therefore the benefit of maintaining L-selectin is not simply a case of licensing T cells to enter the tumor stroma. When L-selectin positive and L-selectin knockout T cells were compared directly in a competitive homing assay, no evidence of L-selectin dependent homing to tumors was observed. A single report describes induction of PNAd on tumor blood vessels in B16 melanomas expressing the surrogate tumor antigen OVA in peritoneal, but not subcutaneously, growing tumors which supports recruitment of naïve L-selectin expressing T cells (33). The absence of PNAd expressing tumor blood vessels and L-selectin dependent homing of T cells in subcutaneously growing NP68-B16 tumors agrees with this published report. Most studies of T cell homing to tumors have focussed on the recruitment of effector T cells into B16 melanomas and shown important roles for P/E-selectin and ICAM-1 on tumor blood vessels as well as CXCR3 ligands (44, 45). Further studies will be required to determine which adhesion molecules and chemokines control the L-selectin independent recruitment of unactivated, therapeutic T cells into solid tumors that our studies have revealed.

An alternative explanation for the beneficial effects of LΔP-selectin expressing T cells is to sustain trafficking to lymph nodes where T cells are licensed by activating, tolerogenic or homeostatic signals which could affect the differentiation and/or survival of F5 LΔP T cells. However, F5 LΔP T cells and F5 L-selectin knockout T cells homed equally well to peripheral lymph nodes in tumor bearing mice at the start of therapy. The loss of L-selectin dependent homing of T cells to peripheral lymph nodes in tumor-bearing mice contrasts sharply with our own findings and published studies using similar competitive homing assays where T cell homing to peripheral LN in both naïve and virus infected mice is exquisitely L-selectin dependent (11, 16, 32, 46). Although not explored further, a likely explanation is induction of MAdCAM-1 on HEV in peripheral LN of tumor bearing mice, which occurs in immunized mice (47). Co-expression of PNAd and MAdCAM-1, which binds both α4β7 integrin and L-selectin on T cells (48, 49), would allow entry of L-selectin expressing and L-selectin knockout T cells into peripheral LN, as we have found. Previous studies have shown that B16F10 melanoma regulate CCL21 dependent T cell recruitment from HEV blood vessels in tumor draining LN but the role of L-selectin was not explored (50).

Although equal numbers of F5LΔP and F5 L-selectin knockout T cells reached solid tumors, the activation of T cells was different. Prior to transfer, unactivated T cells had equally low expression of CD69. However, 18 h after transfer, CD69 expression was upregulated on F5LΔP cells but not on F5L-selectin knockout T cells inside tumors. Although CD69 was upregulated on both F5LΔP cells and F5 L-selectin knockout T cells inside lymph nodes and spleen, CD69 was significantly higher on F5LΔP cells, particularly in the spleen. In non-tumor bearing animals which had been irradiated and vaccinated with peptide, CD69 was equally upregulated on F5LΔP cells and F5 L-selectin knockout T cells in the spleen. Therefore, L-selectin enhanced T cells respond to the presence of a tumor differently to L-selectin knockout T cells and upregulate CD69 both inside the tumor and at sites of antigen presentation and priming in secondary lymphoid organs. This is the first report, to our knowledge, that the combination of tumor load and L-selectin status changes the activation of T cells *in vivo* independently of effects on homing. The steady increase in ^89^Zr in tumors following transfer of L-selectin expressing T cells suggests that T cells continue to be recruited over the first 8 days of therapy, but where incoming T cells were before entering the tumor and whether they are already activated is not possible to determine by PET scanning. Whether CD69 expression is maintained on activated F5LΔP tumor infiltrating T cells throughout the course of the therapy and increases the number of CD8^+^ tumor infiltrating T cells due to retention will be interesting to address. L-selectin does not control the differentiation of CTLs in tumor bearing mice, at least in the lungs, where CD107a expression on transferred T cells was unrelated to L-selectin expression. However, L-selectin controls other aspects of T cell activation as shown by increased numbers of CD44^+^ CD27^+^ “central memory” F5LΔP cells in the lungs of tumor bearing mice as well as Ki67 expression and altered proliferation *ex vivo*. Further studies will be required to fully dissect the mechanism(s) underlying L-selectin dependent control of T cell activation, differentiation and proliferation. Which of these pathways underlies the beneficial effects of F5LΔP T cells in adoptive cell tumor therapies and whether they differ for solid and disseminated tumors will be important to address. Since L-selectin knockout T cells do not control tumor growth yet CD69 is upregulated in secondary lymphoid organs, it is unlikely to be related to events in lymph nodes or spleen. The striking finding that CD69 is not upregulated on L-selectin knockout T cells in solid tumors suggests that the beneficial role of L-selectin is linked to activation of therapeutic T cells inside tumors.

The dominant role of L-selectin in controlling T cell homing has focused attention on its adhesive function. The impact of F5LΔP T cells on tumor growth correlated with increased cell surface expression of L-selectin rather than altered levels of soluble L-selectin. Maintained expression of L-selectin at the T cell surface could prolong binding to accessory cells expressing non-PNAd ligands for L-selectin, such as PSGL-1 (51) or MAdCAM-1 (48), and alter transmission of co-stimulatory signals to T cells resulting in increased expression of CD69. L-selectin dependent upregulation of CD69 was seen in tumors and lymphoid organs suggesting that a ligand-expressing accessory cell is unlikely to be tumor cells but an antigen-presenting cell that matures in the tumor microenvironment and re-locates to lymphoid organs. Although other mechanisms are possible, it is striking that the increased efficacy of F5LΔP T cells against pulmonary influenza infection differs fundamentally from the findings reported here because it is not related to altered T cell activation, differentiation or cytotoxicity but instead depends on increased homing of CTLs to virus-infected lungs (11).

Despite remarkable successes in the field of cancer immunotherapy, many solid tumors remain beyond the reach of this approach. One factor that is believed to be problematic is the recruitment of immune cells (endogenous or exogenous) into the tumor stroma, due to the atypical nature of tumor vasculature (7). In clinical and experimental cancers, the development of PNAd expressing HEV correlates with improved patient outcome (52, 53) and the control of tumor growth (33, 54, 55). L-selectin enhanced T cells might be reasonably hypothesized to have an even greater effect where PNAd-expressing tumor vasculature develop, such as in response to immunotherapies (33, 54, 55). The fact that L-selectin is both shed and gene-silenced in the course of normal T cell activation poses potential challenges for translation of this approach. However, the advent of 2nd generation CAR-T cell therapies which involve multiple modifications, means that the gain-of-function modification used in these studies could be applied to CAR-T cells to broaden their clinical scope. However, monitoring endogenous L-selectin expression on TILs or CAR-T cells may be of immediate clinical benefit in the selection of T cell subsets for administration to cancer patients. The fact that we observed improved control of tumor growth independently of homing, which is associated with alterations in early activation, and works in combination with checkpoint blockade inhibitors makes enhancing L-selectin expression a high-value modification that is likely to improve several aspects of T cell function in the fight against cancer.

## Data Availability

The raw data supporting the conclusions of this manuscript will be made available by the authors, without undue reservation, to any qualified researcher.

## Ethics Statement

All mouse experiments conformed to the British Home Office Regulations [Animal (Scientific Procedures) Act, 1986 (Project Licenses PPL30/3188 and 30/2635 to AA)] and the protocol was approved by the Animal Welfare and Ethical Review Body at Cardiff University.

## Author Contributions

HAW and AA conceived the study. HAW, RRPD, JO, and MA performed experiments with assistance from RNM, MV, and SGR. CM developed methods for, and supervised, ^89^Zr production. SJP, MA, and HA performed ^89^Zr cell labeling, PET/CT imaging, and image analysis. AA supervised the study with critical input from AG. HAW and AA wrote the manuscript with contributions from all authors.

### Conflict of Interest Statement

The authors declare that the research was conducted in the absence of any commercial or financial relationships that could be construed as a potential conflict of interest.

## Footnotes

^1^https://www.taconic.com/phenotypic-data/b6-physiological-data-summary.html
